# A Hybrid CNN–Transformer Approach for Photovoltaic Cell Defect Classification Using Electroluminescence Imaging

**DOI:** 10.3390/s26082450

**Published:** 2026-04-16

**Authors:** Miktat Aktaş, Ferdi Doğan, İbrahim Türkoğlu

**Affiliations:** 1 Software Engineering, GTC Gunes Sanayi ve Ticaret AS, Adiyaman 02040, Türkiye; mkttkts@gmail.com; 2 Software Engineering, Graduate School of Sciences, Fırat University, Elazığ 23119, Türkiye; 3 Computer Engineering, Faculty of Engineering, Adiyaman University, Adiyaman 02040, Türkiye; 4 Software Engineering, Faculty of Technology, Fırat University, Elazığ 23119, Türkiye; iturkoglu@firat.edu.tr

**Keywords:** electroluminescence imaging, photovoltaic cell defect classification, CNN–Transformer, solar cell defect detection, multi-class defect

## Abstract

This study addresses the automatic classification of cells in electroluminescent panel images to detect photovoltaic cell defects. The images used in the study were obtained from a solar panel production line. An original dataset consisting of 37,538 cell images with eight defect classes (Cell-Interconnection, Electrically Insulated Cell Parts, Finger Defect, Material, Microcrack, Multi-Defect, Normal, Visual) was prepared by applying RLSA-based automated cell segmentation enhanced with morphological processing to the photovoltaic panel images. A novel CNN–Transformer model with a self-attention mechanism, called PVELNet, is proposed for classifying defect types. Experimental studies were conducted with 16 deep learning models to compare the proposed model. F1-Score, Precision, Recall, and Accuracy evaluation metrics were used in the experimental study. Furthermore, the Confusion Matrix results obtained from the 16 deep learning models and the proposed PVELNet model are presented. The results were obtained using a relatively balanced dataset prepared for this study. PVELNet achieved 95.71% accuracy, outperforming other models. With 1.79 million parameters and a memory requirement of 46.1 MB, the PVELNet model is relatively lightweight. As a result, it demonstrates the potential to control processes on actual solar panel production lines.

## 1. Introduction

Photovoltaic (PV) systems are widely used for generating energy from the sun. Maintaining production quality is essential for the proper functioning and long-term use of these systems. Long-term reliability in PV systems begins with the quality of the cells. Therefore, it is crucial to inspect the cells on the production line for defects using an electroluminescence (EL) device [1,2,3,4]. Early detection of defects allows for longer-term use of the panels and reduces energy production losses. This leads to increased quality, reliability, and efficiency [5]. One of the most important tools for detecting defects on the cells in PV panels is EL imaging devices. An EL device allows for the visualization of any deficiencies in the cell from an image taken before lamination, enabling immediate intervention. The assembled panel cell images from the EL device are examined cell-by-cell by an expert to check for defects [6].

**Figure 1:**
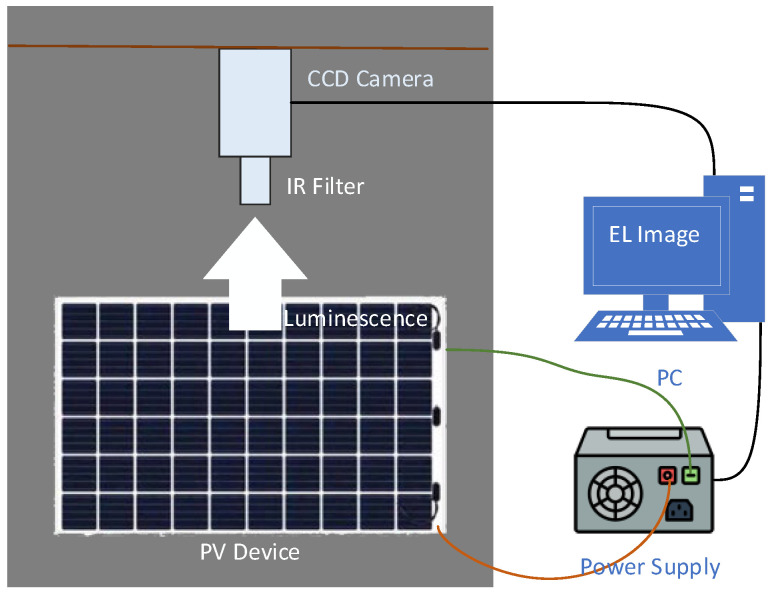
Obtaining panel images using an EL device.

Electroluminescence is a PV panel imaging method used in the solar panel/cell production line to check the quality of solar cells. The device creates an electroluminescence image where defective areas appear dark by applying an external electrical current to the cells [7]. Figure 1 shows a general diagram illustrating the operation of an EL device.

Various studies exist in the literature for determining cell defects and defect types in PV panels [8]. Images taken at the panel level from the EL device are examined to determine whether defects are present by ensuring control in cells. With the development of image processing and deep learning approaches, defects and defect types are automatically classified from cell images [9]. Most studies focus only on whether defects are present in the cell, therefore, they classify cells into only two categories.

The literature remains limited in terms of automatic panel-to-cell segmentation in EL images. Under current conditions, both the cell grid system is adjusted and information such as the number of busbars is entered manually through programs loaded onto the EL device. This situation causes the process to take longer. There is a need for an automated approach that can automatically segment the cells in PV panels to enable separation by cell size and can separate panel cells independently of the number of cells and busbars [10].

The basis of these studies lies in the dataset to be used. There are very few datasets that include various types of defects. The most significant problem encountered in studies conducted in this field is the inadequacy and imbalance of the existing datasets. The datasets currently in use contain datasets for defects, non-defects or a limited number of defect types. In these datasets, the number of images per class is limited [11]. This situation prevents the model from being trained sufficiently in AI-based defect detection systems. In this context, there is a need for cell-based datasets obtained from PV images taken from EL imaging devices [12,13].

In recent years, artificial intelligence-based approaches have become predominant in methods used to detect cell defects and defect types based on EL. There has been a shift towards CNNs (Convolutional Neural Networks), deep learning, Transformers, or hybrid methods in recent years [14,15]. In recent years, studies based on CNNs and transfer learning approaches have been prominent [8,11,16]. An increase in the number of such studies has been observed. There is a need for hybrid approaches that can be used instead of these models.

In addition, many studies have used deep learning models pre-trained on ImageNet. This limits the models’ ability to learn features specific to PV cell defects [17]. Local properties and global dependencies may exist in cells with different types of defects. Pre-trained models will not be sufficient for models to learn this structure. This situation provides motivation for the development of new hybrid approaches.

### Contributions of This Study

Although various studies have been conducted to address the limitations in the literature, many issues still remain unresolved. This article contributes to the detection of damage and its types in PV panel cells by providing the following points:

- Enhanced RLSA (Run Length Smoothing Algorithm)-based cell segmentation: In this study, an enhanced RLSA-based method was applied for the automatic segmentation of PV panels of different sizes. This method enabled the segmentation of 60-cell and 72-cell panels without requiring manual intervention. Cell segmentation was performed on the panel using morphological operations applied after the current RLSA method, and the cells were recorded.
- A balanced new dataset consisting of eight classes: PV panel images taken from an actual production line were used in this study. Approximately 5 million cell images were obtained from 94,000 panel images, and cell labeling was performed in eight different defect classes with the support of experts. These classes are: Fragmented Cell (Electrically Insulated Cell Parts), Material Defective Cell (Material), Finger Defective Cell (Finger), Cell-Interconnection Problem (Cell-Interconnection), Microcrack Cell (Microcrack), Multi-Defect Cell (Multi-Defect), Visual Defect Cell, and Normal Cell. A class-balanced experimental dataset was constructed, with 5000 images for 7 classes and 2538 images for 1 class. This created dataset is more balanced and has a significantly higher number of images than the datasets found in the literature.
- Comprehensive classification comparison with 16 deep learning models: Sixteen models (AlexNet, Vgg16, Vgg19, ResNet18, ResNet50, ResNet101, GoogleNet, InceptionV2, InceptionV3, DenseNet201, MobileNetV2, ShuffleNet, SqueezeNet, DarkNet19, DarkNet53, EfficientNetB0) were used for the classification of cell defects and defect types. The results obtained from the models were analyzed using comprehensive metrics such as Precision, Recall, F1-Score, and Confusion Matrix for comparison. The strengths and weaknesses of the architectures, computational complexity, and costs were compared.
- A New Hybrid Deep Learning Model: PVELNet: The PVELNet model, a novel and original hybrid deep learning architecture designed to classify defects and defect types in PV cells, is presented. The model is a unique architecture that incorporates a CNN–Transformer hybrid structure. By combining the CNN’s ability to learn local features with the Transformer’s self-attention mechanism for learning global dependencies, a new model has been created. The model is a lightweight alternative with a low number of parameters.
- Detailed performance analysis and comparative results: The proposed PVELNet model has been compared in detail with 16 deep learning models. The F1-Score, Recall, Precision, and Confusion Matrix results for each model are provided. Furthermore, the performance of each model in each class is examined in detail according to these evaluation metrics.

This study addresses three fundamental limitations in the literature for multi-class photovoltaic cell defect classification from electroluminescence images. Firstly, there is a lack of EL datasets containing diverse defect classes. Secondly, there is the difficulty of automated panel-to-cell segmentation in EL images with different panel configurations. Thirdly, there is the limited use of hybrid models specific to PV cell defect detection. To overcome these fundamental limitations, an eight-class dataset was created from EL images taken from a real production line, enhanced RLSA-based panel-to-cell segmentation pipeline was proposed, and a lightweight hybrid PVELNet architecture was developed for multi-class defect classification.

## 2. Related Works

Various studies on defect detection and classification of PV cells have been conducted. Upon reviewing these studies, it is observed that there are relatively insufficient defect classes and class sample counts in the datasets [18,19]. Although some larger datasets exist, they are not sufficient in terms of class imbalance and defect types [20,21]. However, a review of the studies reveals that CNN and deep-learning-based approaches are predominantly used for defect classification. It is evident that new and hybrid approaches for defect classification have not been sufficiently explored.

### 2.1. Datasets and Methods in the Literature

The most striking issue in defect types and classification studies is the lack of sufficient datasets. In previous studies, the datasets used consisted of defect classes such as present/absent or a few classes. Wiliani et al. proposed a new dataset consisting of four classes. The dataset, which includes cracks, scratches, stains, and normal cell images, contains 4000 cell images. In the study, tissue feature extraction methods were used to attempt to detect cell defects [18]. In the dataset proposed by Ariffin et al., a dataset consisting of 1437 images containing contact defects, cracks, and connection-problematic cell images from three classes is presented. These images were classified using efficient deep learning models [19]. Another dataset, presented by Imak et al., consists of 2624 cell images, comprising both defective and functional images. In this study, classification was performed using a CNN-based CNN-PCA-SVM hybrid model [22]. One of the large datasets in the literature is the dataset prepared by Su et al., consisting of 36,543 images in 10 classes. Although this dataset appears to be a large-scale dataset, there are serious imbalances between classes. The labeled defects points in the dataset were analyzed using YOLO (You Only Look Once) object detection to identify the defects location and class [20]. The analyses performed on the datasets and the important comparative information extracted are detailed in Table 1.

The use of preprocessing techniques is recommended to improve performance in defect detection studies. Examples of these methods include scaling, noise reduction, and dimensional shift, which are commonly used to analyze images. Furthermore, data augmentation is recommended using methods such as rotation, translation, and reflection to address class imbalances [25,26]. Traditional machine learning techniques are also used in classification studies. Algorithms such as KAZE, SIFT, and SURF are preferred for feature extraction in these studies. These methods show lower performance compared to methods such as CNN. As an example, a study achieved 72.74% performance [27]. Alongside these studies, AI-based CNN approaches are also present. Deep learning architectures such as InceptionV3, Vgg16, and Vgg19 are used for defect classes. These studies report a performance of 93.59% [28]. Studies conducted on some datasets indicate that the Vgg16 and Vgg19 deep learning architectures perform better [29]. In another study on defect classification, SqueezeNet and ShuffleNet architectures were used, achieving 0.92 Precision, Recall, and F1-Score [30]. Another study indicated that SqueezeNet performed more accurately than other methods, with an improvement of up to 6.26% [31].

In recent years, the number of Transformer-based, attention-based, and various hybrid approaches for detecting PV cell defects has increased [32,33]. Khandagale and Patil performed defect detection using ViT models in their study. They stated that ViT models performed better than the deep learning models used, achieving 94.53% accuracy. Their study was conducted on a dataset containing micro-defects [16]. Manandhar et al. reported that a hybrid model using a hybrid framework combining decision trees and deep learning achieved 97% accuracy for the classification of micro-defects in the dataset [34]. Alanazi et al. stated that they achieved 99% performance from 885 images and five classes using a multi-path CNN architecture integration with a dual attention mechanism. The study used a dataset that also included damage from the external environment [35]. Niu et al. presented a multi-scale hybrid approach with a mixed attention mechanism. The study used four types of defects and emphasized that it achieved high performance [36].

### 2.2. Systematic Analysis of the Literature

Identifying the strengths and weaknesses of current approaches to PV cell defects detection and classification provides guidance for future studies. A systematic analysis of the literature is necessary to determine the limitations in this area. A systematic analysis of the studies in the literature in terms of dataset and size, imaging modality coverage, class distribution balance, study quality and standards, and data augmentation applications is presented in Table 2. This analysis is presented by examining the observed differences and their causes in the context of common findings in the literature.

Table 2 details the insufficient sample sizes in the existing datasets, the small number of classes in the datasets, the scope of cell imaging systems, the quality and standards of the images obtained, and the data augmentation strategies applied to eliminate class imbalance in low-numbered datasets.

### 2.3. Interim Evaluation and Motivation for the Work

Table 1 and Table 2 contain a detailed literature review. These literature studies reveal that some limitations and deficiencies still persist and that there are issues that need to be addressed. The foremost of these problems are those related to the dataset. In the literature, dataset insufficiency or low class numbers in the dataset and data imbalance in classes are seen as significant problems. Low defect classes are an important factor limiting generalizability to actual production lines. Furthermore, the low number of data samples in the classes within the dataset negatively impacts the training of the model that detects the defect types and class. The model is insufficient in its training and testing processes.

Studies show that CNN-based deep learning models are mostly used in defects classification and detection studies. Many studies in this field indicate that CNN-based approaches have reached saturation. The classification of defects and its type in PV cells presents unique challenges, as defect classes are similar. Therefore, it has been observed that the number of models or approaches specifically developed for the classification of PV cell defect is quite limited.

The emergence of the problems mentioned above is the most important motivation for this study. In this study, a dataset consisting of 37,538 balanced images from eight classes was created. This dataset is quite rich in terms of class diversity and sample size. Furthermore, this dataset represents an important dataset obtained from a real production environment. Furthermore, the PVELNet model, a specially designed hybrid model for detecting this type of defect, is proposed. This model is a hybrid model that utilizes the local features of CNN and the attention mechanism of the Transformer model to reveal global dependencies. Considering the limitations in the literature, this study is expected to contribute to the field.

## 3. Materials and Methods

### 3.1. Acquisition of the Dataset

**Figure 2:**
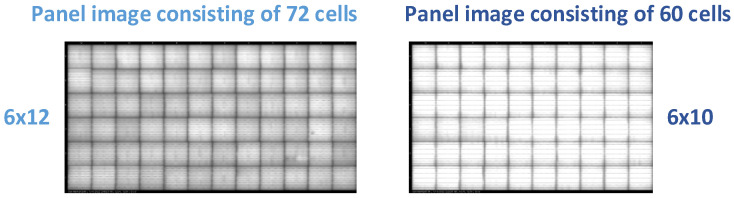
Panel images of 60-cell and 72-cell samples measuring 1992 × 1000 mm obtained from the EL device.

EL images were acquired in a dark imaging booth, as required by its nature. The electroluminescence emitted by the cells, applied with direct current to the panels, was captured by high-resolution CCD cameras. Approximately 94,000 EL panel images were obtained from a local panel manufacturing company. The images were captured on an actual production line during quality control processes. Standard EL test procedures were applied to the images before and after lamination. The panel images shown in Figure 2 are examples of the images obtained. The dataset includes two types of photovoltaic panels: 60-cell and 72-cell panels. In the 60-cell panel, each cell measured 156.75 × 156.75 mm, and the modules featured a four-busbar configuration. In the 72-cell panel, each cell measures 158.75 × 158.75 mm and has a five-busbar configuration. The physical dimensions of the panels are 1992 × 1000 mm.

These images have different busbar counts, such as 4, 5 and 9. Images consisting of 60-cell and 72-cell panels, respectively, are available in different resolutions, such as 4811 × 2967, 4841 × 2979, 5902 × 3048, etc. It has been determined that the images obtained from the EL device are not suitable for certain technical reasons.

This section covers the image processing procedures based on panels. The formation of the cell-based dataset is detailed in the following section.

### 3.2. Segmenting Panel Images into Cells

Defects in panel images obtained from the EL imaging device are cell-based and localized. Defects in the cells on the panel should be evaluated on a cell-by-cell basis. To identify cell-based defect images, the cells on the panel must be separated. Classifying the cells on the panels into categories, defining defect classes, and labeling them is crucial for the study to progress correctly. The cells in each panel image must be separated to obtain a cell image. Therefore, the RLSA algorithm was used to automatically separate the panel images into cells. The applied method was developed for cell segmentation to ensure that the cells were completely extracted. The panels feature 156.75 × 156.75 mm cells in 60-cell modules and 158.75 × 158.75 mm cells in 72-cell modules. The panel dimensions are 1992 × 1000 mm.

RLSA is a classical image processing technique. It enhances structural continuity by closing short gaps between adjacent foreground regions. In EL images, it is useful for strengthening the continuity of cell regions in a panel and reducing fragmentation in the binary representation. When applied alone, classical RLSA can lead to merged or incomplete cell regions due to irregular illumination, weak boundaries, and residual noise. Therefore, classical RLSA is insufficient for reliable panel-to-cell separation. Consequently, in this study, an improved RLSA-based segmentation pipeline was developed by combining classical RLSA with small-object removal, morphological closing, connected component analysis, and ROI-based cell extraction.

**Figure 3:**
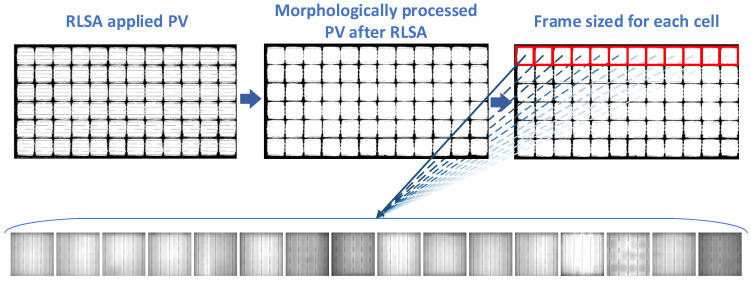
Cell segmentation of 60-cell (156.75 × 156.75 mm) and 72-cell (158.75 × 158.75 mm) photovoltaic panels using enhanced RLSA.

The RLSA algorithm is an image processing technique used to correct text curves in text and document files, and which is used to extract text [52]. The aim was to perform cell segmentation of panel images using this algorithm. As seen in Figure 3, the PV image with RLSA applied did not provide the desired segmentation. The cell boundaries on the panel were strengthened using morphological closing (dilation followed by erosion) operations. After segmentation, each cell image became more suitable. Thus, small objects in the image were eliminated, and the empty busbars were filled. The goal was to obtain a single cell image.

In the developed RLSA segmentation process, cell continuity is strengthened, while subsequent morphological and region-based operations improve cell boundaries. This allows for the systematic extraction of individual cell images. Figure 3 shows that in the final stage, the initial coordinates of the cell in the PV image are determined, enabling subsequent cells to be extracted by shifting other cells based on the dimensions obtained from these coordinates. Each cell in the panel images has the same dimensions. Rather than finding the dimensions of each cell, determining the dimensions of the first cell is crucial for estimating the dimensions of subsequent cells. Finding the first pixel on the X1, Y1 axis of the first cell in the panel image and the X2, Y2 point, which is the end point of the first cell, determines the dimensions of the cells. Then, this first cell frame is moved around the panel to extract the cells. Connected component analysis was used to detect each cell region, which was then geometrically separated and cut using ROI (Region of Interest) logic. Figure 3 illustrates the visual stages of the enhanced RLSA-based segmentation process, while Algorithm 1 provides the corresponding step-by-step computational workflow.

The segmentation process in this study is based on an RLSA-based approach, supported by enhanced RLSA methods using image processing techniques. The EL image is converted into a grayscale binary image for cell region and background extraction. RLSA was applied to highlight horizontal and vertical continuity. Since this algorithm was insufficient, additional enhancement steps were added. Filtering was applied to eliminate small extracellular components and discontinuities in the image. Morphological closure was applied to strengthen cell boundaries.

In the application performed with this approach, the structural integrity of the regions in the segmented panel images was significantly improved. Subsequently, connected component analysis is used to identify candidate cell regions. Instead of identifying each cell independently, the first valid cell region is determined and its bounding box coordinates (X1, Y1, X2, Y2) are calculated. Based on this reference cell, the remaining cells are automatically extracted using a grid spread strategy that takes advantage of the repeating geometric structure of the PV panels.
**Algorithm 1:** Enhanced RLSA-Based Segmentation for Panel-to-Cell ExtractionInput:    EL panel image I Output:    Set of segmented cell images {C1, C2, …, Cn} 1: Convert I to grayscale image Ig 2: Apply thresholding to obtain binary image Ib 3: Apply Run-Length Smoothing Algorithm (RLSA) on Ib to enhance horizontal and vertical continuity 4: Remove small non-cell regions using area-based filtering (e.g., bwareaopen) 5:   Apply morphological closing (dilation followed by erosion) to strengthen fragmented cell boundaries 6:   Perform connected component labeling on the refined image 7: Identify candidate cell regions based on geometric constraints 8: Select the first valid cell region and determine its bounding box: (x1, y1, x2, y2) 9: Compute cell width (w) and height (h) from the bounding box 10: Initialize grid-based propagation using (w, h) 11: For each grid position across the panel image do 12: Define ROI using:    x = x1 + k × w    y = y1 + m × h 13:  Extract ROI as candidate cell image Ci 14:  Validate ROI based on boundary clarity and size constraints 15:  If valid: 16:    Store Ci with panel ID and cell index 17:  Else: 18:    Discard ROI 19: End For 20: Return all valid segmented cell imageslgorithm 1. Enhanced RLSA-Based Segmentation for Panel-to-Cell Extraction

Each detected region is cropped as an ROI and stored as a separate cell image using panel-based indexing. To ensure dataset quality, regions with unclear boundaries or insufficient structural integrity are excluded. This ensures robust cell extraction across panels with varying resolutions and structural variations.

To quantitatively evaluate the performance of the segmentation process, the proposed improved RLSA-based method was tested on 1000 panel images. The cell segmentation success rate of the panel was measured at 97.1%. Examination of cell and panel images where segmentation failed revealed that these were mostly associated with image acquisition problems such as low contrast, uneven lighting, blurring, sensor noise, and partially cropped panels. These examples were excluded during dataset preparation to maintain the reliability of the segmented cell dataset.

The cell sizes obtained from panel images with different resolutions also vary in size. Cell images ranging from a minimum size of 141 × 143 to a maximum size of 948 × 950 were obtained. Images where cell edges were not clearly defined were excluded.

### 3.3. Cell Defect Classification

Approximately 5 million cell images of various sizes were obtained. It was determined that the vast majority of the images obtained were normal cell images. The obtained cell images were organized into eight classes with the assistance of experts. The eight classes in the dataset prepared for this study were selected based on specific criteria. These classes represent distinguishable visual patterns observed in industrial EL images. They were selected to practically represent relevant cell conditions. Three criteria were used when determining the classes: (i) relevance to actual production and quality-control processes, (ii) consistency with defect types commonly reported in the literature, and (iii) the inclusion of a sufficient number of images to ensure adequate representation in the existing image pool. To specifically reflect structural and electrical failures in these defect types, physically meaningful defect classes such as Microcracks, finger-related defects, material-related defects, and Cell-Interconnection defects were included. The Visual class was defined to represent imaging artifacts identified through expert evaluation. Additionally, the Multi-Defect class was included to represent cells containing multiple coexisting defect types within a single cell and cannot be assigned to a single class. These defect classes, consisting of Cell-Interconnection, Electrically_Insulated_Cell_parts, Finger, Material, Microcrack, Multi-Defect, Normal, and Visual, and the defect class information are detailed below. The primary causes of the eight defect classes, their EL image characteristics, and their expected performance effects are summarized in Table 3.

**Figure 4:**
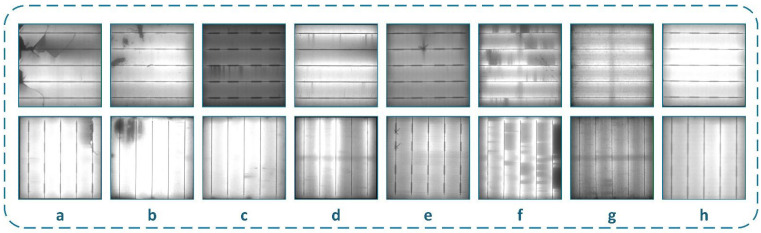
Cell images for each class. (**a**) Electrically Insulated Cell Parts; (**b**) Material Defect; (**c**) Finger Defect; (**d**) Cell-Interconnection; (**e**) Microcrack; (**f**) Multi-Defect; (**g**) Visual; (**h**) Normal.

These defect-class definitions were developed with expert assistance during the data preparation phase. The labeling process was carried out with expert support. It was based on a joint evaluation by four of the eight engineers working on the production line. Three of the experts were electrical/electronics engineers, and one was a mechanical engineer. The experts had practical experience in industrial inspection processes. Class labels were determined through consensus-based evaluation. A formal inter-annotator agreement metric was not computed. However, ambiguous image samples were jointly reviewed by the experts until a common decision was reached. Sample images for these eight classes are provided in Figure 4.

### 3.4. Data Distribution and Data Augmentation

The extracted cell images were labeled by class and saved. Various numbers of images have been obtained in the class distributions associated with these images. The classes with a high number of images are Finger, Material, Microcrack, Multi-Defect, and Normal. A limit of 5000 cell images was randomly selected from the images in these classes to create the dataset. The class distributions of the images in the created dataset are given in Table 4.

To ensure that classes with a low number of images in the dataset are balanced, the images were replicated using data augmentation methods. The following methods were used for data augmentation: 90-degree rotation, 180-degree rotation, 270-degree rotation, reflection on the x-axis, and reflection on the y-axis. These methods were applied to images belonging to the Cell-Interconnection, Electrically_Insulated_Cell_parts, and Visual classes (augmentation was stopped when 5000 images were completed). The class-wise image counts after data augmentation are provided in Table 5.

Table 5 shows the class-based distribution of training and test images before and after data augmentation. The dataset was first divided into training and test subsets. Data augmentation was only applied to the training data for underrepresented classes. Thus, a total of 37,538 images were used in the training and testing processes. No data augmentation was applied to the test set. This approach reduced class imbalance while providing an unbiased assessment on previously unseen samples. Although data augmentation methods have improved the representation of classes with small sample sizes, overly synthetic transformations can lead to augmentation-related bias. Therefore, augmentation operations have been limited to realistic geometric operations and applied only in training classes with small sample sizes.

### 3.5. Used Deep Learning Models

In this study, 16 different pre-trained deep learning models were used to classify the eight-class cell defect images created. Each model used was previously trained on the ImageNet dataset. It was adapted for cell classification using the transfer learning approach. The deep learning models used were DarkNet19, DarkNet53, MobileNetV2, ResNet18, ResNet50, ResNet101, Vgg16, Vgg19, SqueezeNet, GoogleNet, DenseNet201, AlexNet, InceptionV3, EfficientNetb0, InceptionV2, and ShuffleNet. Different models were used to compare the performance of the proposed model with models that have different layers, depths, and numbers of parameters. These models are categorized according to their architectures in Table 6.

Sixteen fundamental deep learning models—including classical CNNs, convolutional networks, densely connected models, inception-based designs, lightweight/mobile architectures, and scalable backbones—have been selected to evaluate their performance on classification tasks across different architectural families. The models demonstrate their capabilities in challenging classes such as Microcracks, Multiple Defects, Finger Defects, and Electrically Insulated Cell Parts. Easier classes, such as Normal, yield higher and more stable results across most architectures. Consequently, the models were also used to analyze both accuracy comparisons and the robustness of different defect types at the class level. The technical specifications of each deep learning model are provided in Table 7.

**Figure 5:**
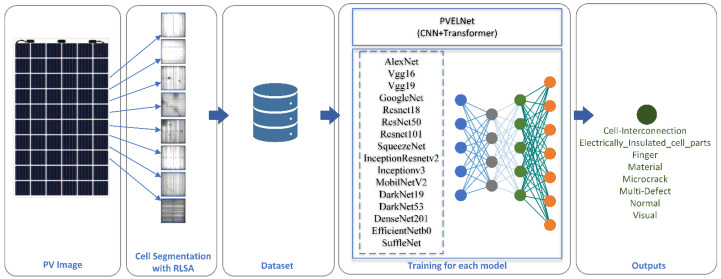
The implementation process steps of the study.

Table 7 lists the deep learning models used and their technical specifications. The hyperparameters used for the deep learning models and the proposed PVELNet model are provided in Table 8. The same hyperparameters were used to ensure a fair comparative analysis of the models and the proposed model. The overall architecture of the study is shown in Figure 5. The deep learning models used and the process steps for implementing the proposed model are illustrated in the figure. The goal was to standardize hyperparameter selection across all models by evaluating them using the same data split, the same optimizer, the same batch size, and the same number of epochs.

All experiments were conducted on a workstation equipped with an Intel Core İ9-14900 processor, 48 GB RAM, and an NVIDIA RTX 4080 GPU, using MATLAB (R2024b, MathWorks).

The overall architecture of the study is shown in Figure 5. The deep learning models used and the process steps for implementing the proposed model are illustrated in the figure.

In the study, panel images were first obtained. Subsequently, the RLSA segmentation technique was developed and applied to separate the panel images into cells (segmentation). After the cells were segmented, each cell was assigned to one of the eight classes in the dataset by experts. Classes with very few and very many instances in the dataset were adjusted. The maximum number of instances for classes with many instances was set to 5000. For images belonging to classes with few instances, data augmentation was performed to create a balanced dataset. After the dataset was prepared, deep learning models were trained and tested using transfer learning with the same hyperparameters. The proposed PVELNet model was also used in this training and testing process. Since other models were used with transfer learning, the PVELNet model was trained twice to be evaluated under equal conditions. The test results obtained from the models were analyzed using evaluation metrics, and the results are presented.

### 3.6. Recommended Model: PVELNet

The proposed PVELNet model is based on the visual characteristics of photovoltaic cell defects observed in EL images. Some defect types, such as Microcracks, finger-shaped interruptions, and interconnection-related defects, appear as subtle local structural patterns, weak line discontinuities, or small dark regions requiring detailed local feature extraction. In contrast, other classes, such as material-related defects, visual defects, and multiple defect cases, may involve broader spatial distributions, contextual irregularities, or the coexistence of multiple patterns within the same cell. For these reasons, PVELNet was designed, incorporating multi-scale convolutional branches for local defect representation. It is also designed as a task-specific hybrid architecture that combines Transformer-based global modeling to capture broader spatial dependencies in EL cell images.

The proposed architecture aims to capture fine-scale defect cues such as parallel convolutional branches, subtle crack traces, finger-like discontinuities, and localized intensity changes. Residual and concatenation paths in the architecture help preserve weak but distinctive defect-indicating patterns. Spatial-to-sequence transformation and positional encoding are included to preserve the structural arrangement of cell regions prior to Transformer-based processing. With these approaches, the specified features in the model enable it to analyze the details of local defects and broader contextual relationships that are important for heterogeneous classes such as Material and Multi-Defect.

Unlike general CNN–Transformer hybrids, the proposed hybrid PVELNet model is specifically designed for photovoltaic cell defect classification. This innovation lies in the model’s integration of multi-scale parallel convolutional branches for fine-grained local defect extraction, residual/addition and depth-concatenation paths for feature preservation and enhancement, and a spatial-to-sequence transformation with positional encoding to preserve defect location information prior to Transformer-based global modeling. In addition to integrating local and global representations, PVELNet is adapted to address the heterogeneous visual structure of PV cell defects, such as Microcracks, finger defects, darkened regions, and multiple defect patterns.

This study proposes a new model called PVELNet for classifying defects and defect types of cells in PV panels. It is a hybrid model based on CNN features that enable local feature extraction and a Transformer (self-attention) that can extract global features. The input layer of the model contains a 256 × 256 input. To distinguish classes in images of defective cells, the model applies convolution operations of 1 × 1, 3 × 3, 5 × 5, and 7 × 7 dimensions in parallel branches using the inception module. These convolution operations help to more clearly distinguish cracks, finger defects, darkened areas, separations, and regional differences. These features are integrated using depth concatenation and residual/addition layers. The integration aims to prevent information loss and eliminate the gradient vanishing problem. Batch normalization, ReLU (Rectified Linear Unit), and LeakyReLU activation functions were used throughout the model. This ensured a more stable path during model training. It demonstrated non-linear representational power. The model reveals its representational power by extracting feature maps. The extraction of local and discriminative features in the cell has a positive effect on evaluation metrics in classification. Distinctive features are extracted from feature maps and then vectorized into token sequences via spatial-to-sequence transformation. The vectorized sequence enables the transition from the convolutional structure to Transformer-based sequential learning. Token sequences ensure the preservation of spatial information. For this purpose, sinusoidal positional encoding is added to the token. Thus, the Transformer layers attempt to learn by also considering the positional information of defective cells.

The Transformer encoder block consists of multi-head self-attention and fully connected layers. Each sub-block here is supported by residual connections and layer normalization. This enables the learning of connections and relationships between cell regions. The token obtained at the Transformer output is summarized using one-dimensional global average pooling. Then, a fully connected layer and a classification layer are used to generate the probability value of the image belonging to eight different defect classes.

The proposed PVELNet model is a new deep hybrid model created by combining CNN and Transformer structures. The model has the ability to use local and global features simultaneously. Thus, it can provide high accuracy and generalizability in defect classification.

**Figure 6:**
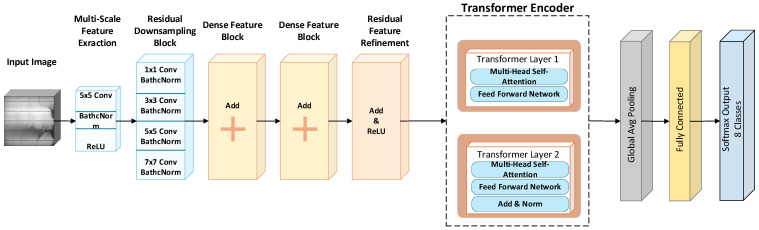
PVELNet model architecture.

In this study, panel images are obtained from the EL device, and then PV images are segmented at the cell level to separate the cells. The separated cells are trained using the developed hybrid deep learning model, and classification is performed. Figure 6 shows the architectural view of the model.

The PVELNet model proposed in this study is specifically designed for detecting/classification PV cell defects. The model is an innovative hybrid model that combines CNN and Transformer attention. With a 256 × 256 × 3 dimensional input, the model categorizes defects into eight classes for output. The model incorporates multi-scale feature extraction, residual downsampling blocks, dense and residual feature refinement, and transfer encoder classifier blocks.

Filtering occurs with a 5 × 5 convolution layer after PVELNet’s input layer. This layer enables the formation of basic feature maps in the image. Subsequently, parallel paths inspired by the inception architecture are present. Four different convolution operations of 1 × 1, 3 × 3, 5 × 5, and 7 × 7 dimensions are applied in parallel simultaneously. This captures local and global information in the image. Each parallel path contains activation and normalization layers. These layers are combined with the summation layer. This structure facilitates the detection of defect of different sizes.

Residual connections and depth concatenation techniques are used within the model to enrich and deepen the feature maps. This layered structure contains 32-filter convolution layers. These convolution layers increase the feature size, and spatial resolution is reduced using stride-2 sampling. This aims to improve computational efficiency. Multiple convolution and activation layers are arranged in parallel blocks. Parallel blocks ensure increased feature diversity. The outputs of the blocks are combined using depth concatenation. The model learns different abstraction levels. Batch normalization and dropout layers are used to reduce training time and the risk of overfitting.

The transition point from the CNN architecture to the Transformer architecture is the Transformer Encoder section shown in Figure 6. Here, feature maps are converted into a sequence order with a spatial structure using the SpatialToSequence function. The 2D feature map is converted into a 1D token sequence. Then, sinusoidal positional encoding is added to ensure that the Transformer regains the spatial position information it lost. This conversion allows visual features to be processed like word sequences in language models.

The final section of the model contains two transformer encoders. Each block has a multi-head self-attention and feed-forward network layer. The self-attention mechanism in the model allows the connection between the start and end of the defect to be weighted. For example, weighting is applied between the starting point of a fracture and the point where the fracture ends. This structure reveals global dependencies that CNNs cannot capture. A fully connected layer is placed after each attention and feed-forward layer. Then, gradient refinement is performed by applying layer normalization. In the final part of the model, a global average pooling layer converts the data into a single-vector sequence. Classification is then performed using a fully connected layer with eight classes and softmax activation.

The fundamental feature of the proposed model is the combination of CNN’s powerful local feature extraction with Transformers’ modeling of global dependencies. This enables PVELNet to detect both small abnormalities within the cell and the distribution of these abnormalities. It also allows for the simultaneous evaluation of the relationship between multiple defects that may occur within the cell.

### 3.7. Evaluation Metrics

The evaluation metrics commonly used in the literature have been included to assess the results obtained from 16 different deep learning models and the proposed hybrid deep learning model. These metrics have been used for each deep learning model and the proposed PVELNet hybrid model. The metrics are critical for comparing and analyzing the results. Providing only accuracy from the evaluation metrics may not be a sufficient metric. The similarity of defect types and minor deficiencies in class distribution make it even more necessary to examine performance. Therefore, for a comprehensive comparison, the F1-Score, Accuracy, Precision, and Recall metrics were evaluated together. Precision is used to measure the effect of false positives. Recall indicates the success in capturing examples belonging to the relevant class. The F1-Score offers a separate evaluation opportunity as the harmonic mean of Precision and Recall. Table 9 contains the equations for the evaluation metrics and explanations of the metrics.

The Confusion Matrix is an evaluation method used to compare results with other classes. It is particularly used to analyze the difficulty of classification between classes with similar visual characteristics.

## 4. Experimental Results

### 4.1. Comparison of Overall Performances

The results obtained in the study are presented according to the evaluation metrics F1-Score, Precision, Recall, and Accuracy. When the results obtained were evaluated as F1-Score, it was observed that the results ranged from 0.83 to 1. The F1-Score values for the eight classes are shown in Table 10 for each model. Upon examining this table, it is seen that results in the range of 0.94–0.98 were obtained for Cell-Interconnection, 0.94–0.98 for Electrically Insulated Cell Parts, 0.84–0.93 for Finger, 0.86–0.93 for Material, 0.83–0.92 for Microcrack, 0.95–0.97 for Multi-Defect, 0.98–1 for Normal, and 0.91–0.97 for Visual. According to the F1-Score, the model with the highest mean F1-Score was the recommended PVELNet model with 0.96. Among 16 deep learning models, the best average F1-Score was produced by DarkNet53, Vgg16, and Vgg19 with 0.95. The lowest average F1-Score was produced by EfficientNetB0.

When looking at defect classes, the detection of normal cell images is quite high. Distinguishing itself from other defect classes, it has performed quite successfully for almost all models. Microcrack cells showed the lowest performance among the defect classes. In the classification of images in this class, F1-Scores in the range of 0.83–0.92 were generally produced. The best result in detecting this class of defect was found by PVELNet with 0.92. In practice, images belonging to the Microcrack and Multi-Defect classes, which have the highest probability of confusion, achieved strong scores of 0.92 and 0.97 with the proposed model.

Class-based performance should be interpreted more carefully, as physical defect types, image quality-related classes, and multiple defects all involve images with different characteristics. Therefore, some of the variation in observed classification performance may also be influenced by the structure of the class taxonomy.

Table 10 shows the F1-Score values of deep learning models for each class. In addition to this evaluation metric, data including Recall and Precision values for a different evaluation of both models and classes are provided in Table 11 and Table 12.

**Figure 7:**
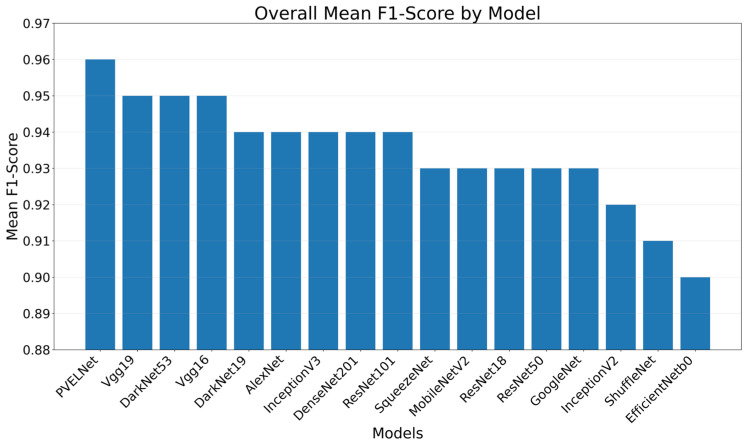
Average F1-Score results for the models.

A bar chart showing the average F1-Score values for each model is presented in Figure 7.

When evaluating the results obtained based on Precision and Recall values, Precision values between 0.80 and 1 and Recall values between 0.80 and 1 were observed. The Recall values for the eight classes are listed in Table 11 for each model. Upon examining Table 11, Cell-Interconnection ranges from 0.90 to 0.97, Electrically Insulated Cell Parts from 0.90 to 0.99, Finger from 0.81 to 0. 97, Material from 0.85 to 0.95, Microcrack from 0.80 to 0.92, Multi-Defect from 0.97 to 0.99, Normal from 0.98 to 1, and Visual from 0.91 to 0.97. High Recall values indicate that the models will not miss defective images, showing a reduced false-negative rate. The model with the highest mean Recall value was the recommended PVELNet model with 0.96. Among the 16 deep learning models, the best average Recall was produced by DarkNet53, Vgg16, and Vgg19 with 0.95. The lowest average Recall was produced by EfficientNetB0, at 0.90.

Looking at the Recall values for defect classes, the detection of normal cell images is quite high. It stands out from other defect classes and shows a highly successful performance by almost all models. The lowest performance among the defect classes was shown by Microcrack cells, at 0.87. In general, Recall values between 0.83 and 0.92 were produced for the classification of images in this class. The best result for detecting defect in this class was found by PVELNet with 0.92. This makes it more feasible for implementation on the production line.

**Figure 8:**
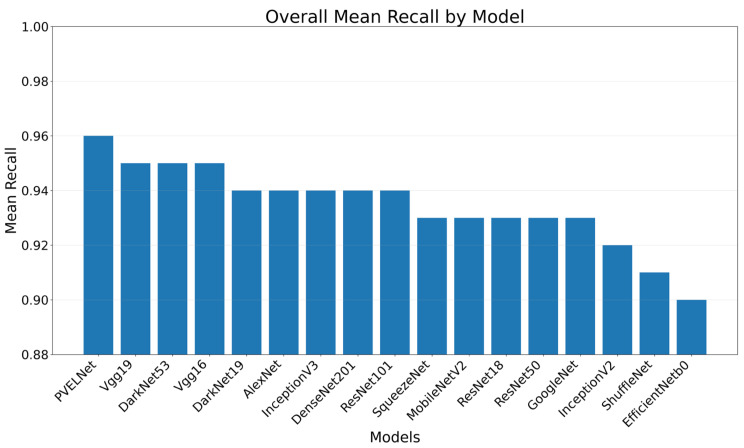
Average Recall results for the models.

A bar chart showing the average Recall values for each model is presented in Figure 8.

Table 12 presents the Precision values for the models. Cell-Interconnection ranges from 0.91 to 0.98, Electrically Insulated Cell Parts from 0.95 to 0.98, Finger from 0.85 to 0.93, Material from 0.84 to 0.93, Microcrack from 0.80 to 0.92, Multi-Defect from 0.94 to 0.98, Normal 0.99–1, and Visual 0.88–0.96. The mean model with the best Precision value was the recommended PVELNet model with 0.96. Among the 16 deep learning models, the best average Precision was produced by DarkNet19, DarkNet53, Vgg16, and Vgg19 with 0.95. The lowest average Precision was produced by EfficientNetB0 with 0.90.

Looking at the Precision values of the defect classes, it is seen that the detection of Normal cell images is quite high. It performed quite well compared to other defect classes. The lowest performance among the defect classes was shown by Microcrack cells with 0.88. In general, Precision values between 0.80 and 0.92 were produced in the classification of images in this class. The best result in detecting defects in this class was found by PVELNet with 0.92.

**Figure 9:**
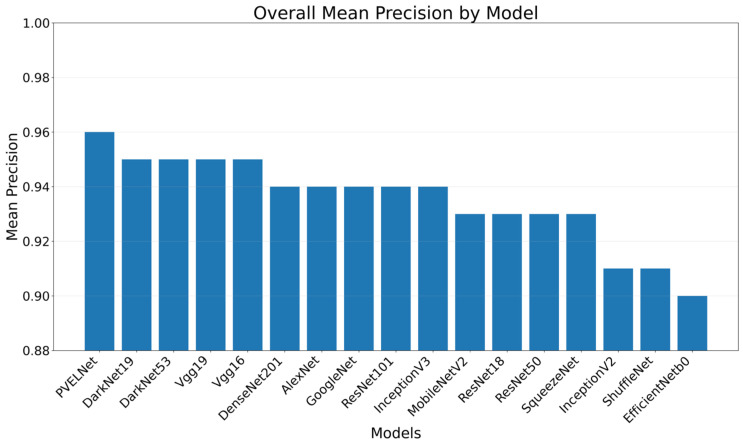
Average Precision results for the models.

A bar chart showing the average Precision values for each model is presented in Figure 9.

The table data for the models’ Recall, Precision, and F1-Score evaluation metrics are provided in detail in Table 10, Table 11 and Table 12. The accuracy data for the correctness of the results in the test data are provided in Table 13. In addition to the accuracy value, the table also provides the disk space occupied by each model after training in megabytes.

When examining the accuracy values, it is seen that the 17 models, including the recommended model, range from 90.02% to 95.71%. Among the models, the best result was achieved by the recommended model PVELNet with 95.71%. Among the other deep learning models besides the recommended model, the highest performance was achieved by Vgg19 with 94.69% and DarkNet53 with 94.65%. The lowest performance rates among the models were 90.02% for EfficientNetb0 and 91.1% for ShuffleNet.

Furthermore, when examining the models, significant differences in computational costs were observed. When examining memory requirements, the models ranged from 13.3 Mb to 1505 Mb. Significant differences were observed in the memory space occupied by the models after training. Here, it can be seen that the number of parameters given in Table 7 corresponds to the space occupied on disk. The models that occupy the least disk space are SqueezeNet, ShuffleNet, MobileNetV2, and PVELNet. Therefore, these are the lightest models among the 17 models.

### 4.2. PVELNet Performance Analysis

The proposed model PVELNet has been evaluated using accuracy as well as class sensitivity metrics such as F1-Score, Precision, and Recall. Although the defect types in EL cell images appear similar, the model demonstrates that it has effectively learned the features associated with the defects in the images. Within the newly created dataset, the model has demonstrated high-level performance based on a generally balanced distribution across classes. Table 10, Table 11, Table 12 and Table 13 show that PVELNet has achieved better Accuracy, F1-Score, Precision, and Recall values than the 16 deep learning models it was compared to. The performance of the PVELNet model has proven to yield balanced and stable results. The model’s F1-Score, Recall, and Precision values range between 0.92 and 1 across classes.

The overall accuracy of PVELNet was 95.71%. This performance yielded a highly successful result when compared to the 16 deep learning models used with transfer learning. It demonstrated balanced performance across all eight defect classes. Considering the error distributions between classes, it performed well even in difficult classes.

According to F1-Score results, PVELNet produced quite successful results in the Finger, Material, and Microcrack classes. In addition, almost all models achieved high performance in the “Normal” class. The Microcrack class, which is the most challenging defect type in the literature, generally had the lowest performance. Compared to other models, PVELNet has the best F1-Score in the Microcrack defect type. This success shows that the model is also effective in local and small defects. Furthermore, its generally high performance in the Multi-Defect class shows that multiple defects are also well captured by the model.

The Precision and Recall results indicate that the proposed model exhibits a balanced approach in detecting defect types. The model’s high Recall values indicate a low false-negative rate for defects. Furthermore, the high Recall values indicate a low false-negative rate. These high performance values support the model’s ability to produce high Recall and Precision values in real-world scenarios.

The results show that the proposed PVELNet model can both extract local features and track global dependencies. The CNN architecture identifies local features, while the Transformer-based self-attention block learns the relationships between defects.

In addition to its high performance values, the model stands out with its low memory requirement and low number of parameters. Having 1.79 million parameters and requiring 7.98 MB of memory demonstrates the model’s lightness. The 46.1 MB memory requirement after training is significantly lower than the average memory requirement of other models. The models with the lowest memory requirements are, in order, SqueezeNet, ShuffleNet, MobileNetV2, and PVELNet.

Statistical confidence analysis was conducted to support the performance analysis obtained in the study. The classification accuracy of the proposed PVELNet model was 95.71%. The 95% confidence interval was calculated based on the test set size. The resulting 95% confidence interval ranged from 95.26% to 96.16%. This indicates the stability of the model performance, and that it is not due to random variation. The confidence interval therefore provides additional evidence that the performance improvement of PVELNet is reliable.

### 4.3. Confusion Matrix Results Analysis

**Figure 10:**
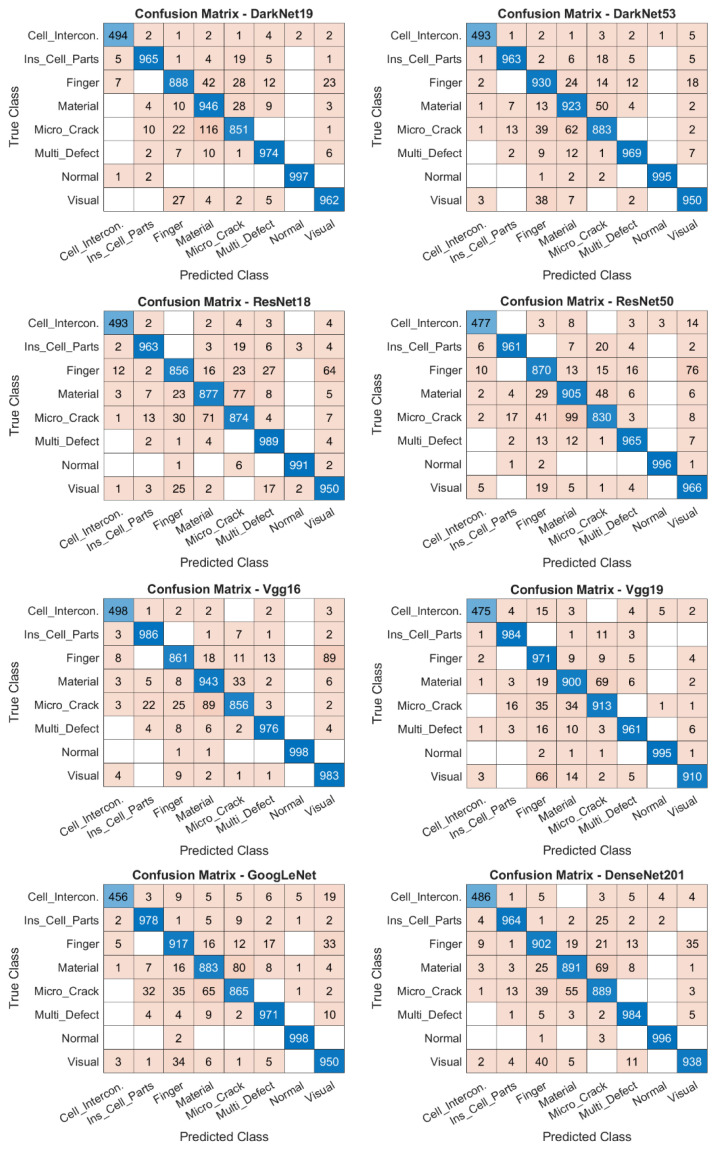

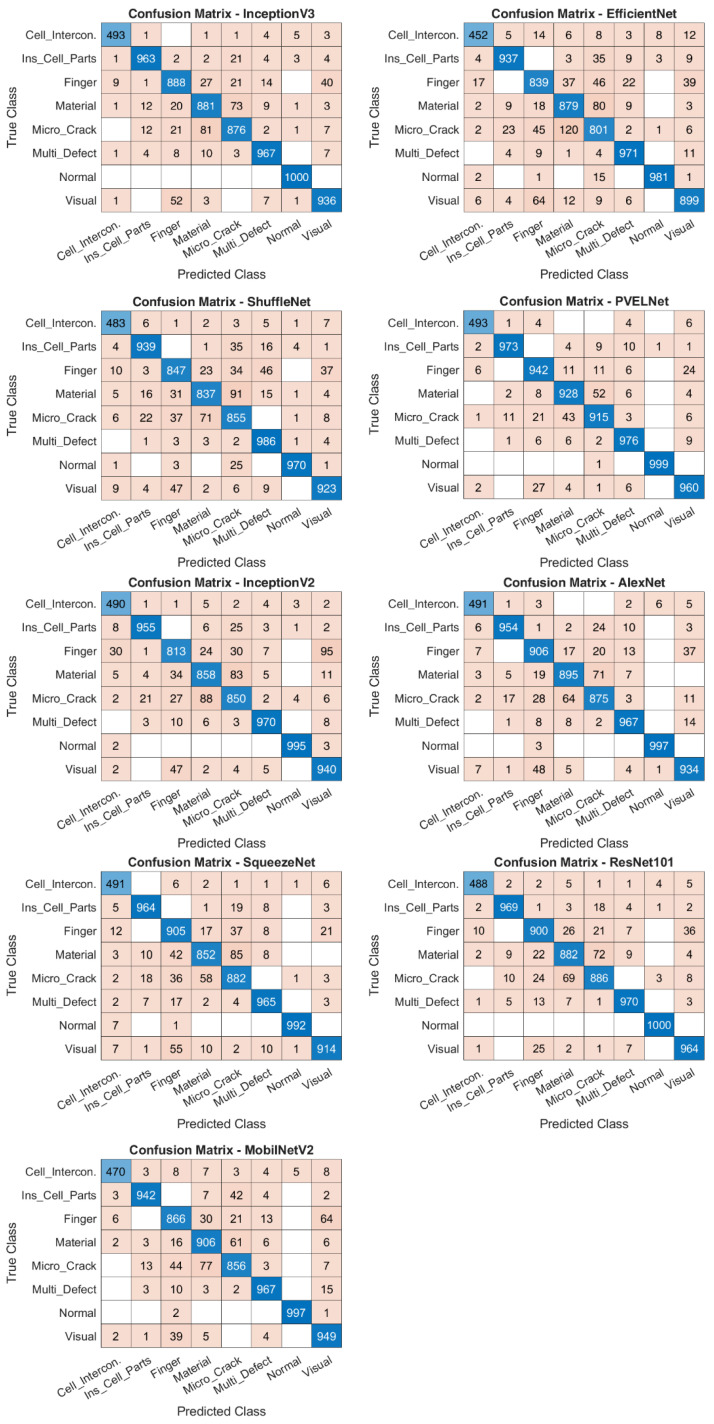
Confusion Matrix for 16 deep learning models and the PVELNet model. Darker cells indicate correctly classified samples, whereas lighter cells indicate misclassified samples.

The Confusion Matrix contains numerical class information showing which errors were made on a class basis. It shows which defect type each class was identified as in the test results, where the defect image for each class was confused with other classes, and also how much of the cell image belonging to the defect class was correctly predicted. Figure 10 shows the results of the 8 × 8 class test images for each model. It provides the class-based correct and incorrect results obtained from each model.

## 5. Discussion

In this study, a novel hybrid CNN + Transformer-based model is proposed for classifying cell defects and defect types in PV panels. To evaluate the performance of the proposed model, 16 pre-trained deep learning models were analyzed using Precision, Recall, and Accuracy evaluation metrics. Additionally, a Confusion Matrix was provided for each model. The accuracy rate obtained from the model was 95.71%, which represents the highest accuracy among the compared models. This accuracy rate produced better results than 16 deep learning models. It shows that it performs successfully in different classes with a 0.92 F1-Score, even for Microcracks, which are among the most difficult defects to classify. It is seen that PVELNet produces quite successful results by combining CNN’s ability to find local features with Transformers’ ability to find global connections thanks to their self-attention mechanism. In other words, while finding Microcracks within a cell, it can understand the complex relationship in the Multi-Defect class, which is a different class.

An important achievement of the study is that it has a balanced dataset with eight classes. A new dataset consisting of 37,538 images has been added to the literature, with 2538 examples in the Cell-Interconnection class and 5000 examples in each of the other classes. Different datasets in the literature have a small number of images and a small number of classes. Large-scale datasets in the literature have class imbalance. Therefore, the dataset used in this study is of critical importance.

When examining the performance of the models, all models achieved high performance on images belonging to the “Normal” class. This indicates that the healthy cell image was well learned by all models and clearly distinguished from the defect classes. The study demonstrated that when used in a real production environment, it can clearly detect defect cells. The defect class with the lowest performance was the Microcrack defect. This defect is relatively more difficult to distinguish due to the fine cracks it creates in the cell, its low contrast, and its similarities to other defects. The reason the PVELNet model can detect this type of defect better than other models is that it can see local and global features simultaneously. On the other hand, among the 16 deep learning models, the EfficientNetb0 model showed the lowest performance in correctly detecting defect types. EfficientNetB0, ShuffleNet, and SqueezeNet showed relatively lower performance among all models in the F1-Score, Precision, Recall, and Accuracy evaluation metrics. After the PVELNet model, the models with the highest performance were Vgg19, DarkNet53, Vgg16, and DarkNet19. The performance of each model used in the study is shown in Table 10, Table 11, Table 12 and Table 13. In addition, the Confusion Matrices for each model are given in Figure 10.

The models used should be evaluated not only in terms of evaluation metrics but also in terms of their parameters and memory usage. In terms of memory usage after training, the lightest models are SqueezeNet at 13.3 MB, ShuffleNet at 19.9 MB, MobileNetV2 at 36.8 MB, and PVELNet at 46.1 MB. Table 13 contains the post-training memory space information for the models. The PVELNet model is seen to be competitive with other models in terms of both the number of parameters it has and its memory requirements. Although the proposed model achieved high performance, its generalization capacity should be interpreted appropriately. The cell images included in the dataset were obtained from a different manufacturer, meaning that the environment was relatively homogeneous. While the proposed model performed well in the test environment where it was evaluated, it has not yet been validated with data from different manufacturers and different EL devices. Furthermore, the model may be sensitive to variations in images with low contrast, uneven lighting conditions, blurring, sensor noise, and cropped panel images. Considering these factors, it is seen as a challenging situation, especially in the segmentation process. The current findings do not demonstrate that it can be fully generalized to real industrial conditions. This should be considered proof of performance for the current scenarios. Therefore, future studies should include validations on datasets obtained from different production environments. As a high-performance and lightweight model, it indicates potential suitability for future production-line applications contributes to successful performance in the actual production line.

Although the study has high contribution potential, it also has some limitations. One of these limitations is that the panel images forming the dataset were obtained from a specific EL device belonging to a single manufacturer. Testing with images obtained from different manufacturers’ EL imaging devices is crucial for generalizability. Another limitation is that the cells were classified in the study. In future studies, it would be appropriate to conduct a study that determines the location and type of defect on the panel without performing cell segmentation. Finally, only geometric transformations were applied in the data augmentation methods used in the dataset used in the study. In the future, using different methods such as GAN or diffusion models or synthetic data generation will further increase the robustness of the study.

## 6. Conclusions

In this study, EL images were used to identify cell defects and defect types in PV panels. Approximately 94,000 EL panel images were collected and segmented using the RLSA algorithm, and the panel images were separated into cells. With expert guidance, these images were classified into eight defect types. A balanced dataset was created with 5000 images for seven defect types and 2538 images for one defect class.

In this study, a hybrid model with a CNN + Transformer self-attention mechanism, specifically designed for classifying cells in PV panels, was developed. According to the experimental results, the proposed PVELNet model achieved 95.71% accuracy and outperformed 16 deep learning models.

With 1.79 million parameters and a post-training size of 46.1 MB, PVELNet remained relatively lightweight within the current comparative setting. The study has significant potential for automated quality control applications in photovoltaic production environments. It aims to reduce manual inspection and expert-based evaluation. By accelerating cell defect assessment, it helps to demonstrate a consistent approach in the decision-making process. In addition, the lightweight structure of the proposed model makes it a candidate for integration into real-time monitoring and industrial decision support systems in the future.

In conclusion, this study proposes a solution for the automation of panel production systems that minimizes the need for expert intervention. Thus, a faster and more generalizable PVELNet model has been developed with minimal human intervention. Furthermore, further validation on data from different manufacturers, EL devices, and imaging conditions is needed before claiming broader generalization.

Future studies plan to create a more generalizable structure using images from EL imaging devices of different manufacturers. Additionally, a system that directly detects defect and its location on the panel image is being considered. Furthermore, the goal for future studies is to directly locate the fault without the need for a segmentation process. Another future research objective is the integration of the model into a real-time monitoring system in an industrial inspection environment.

## Figures and Tables

**Table 1:** Comparison table of datasets used in the literature on cell defects.

Dataset Name	Defect Types	Total Image Count	Used Method	Achieved Success
**Wiliani et al. Dataset [18]**	4 (Cracks, Scratches, Stains, Good)	4000	Tissue Feature Extraction	-
**EfficientNet Dataset [19]**	3 (Contact Defects, Cracks, Related Issues)	1437	EfficientNet (B0–B7)	93.93% Accuracy
**İmak EL Dataset [22]**	2 (Defective, Functional)	2624	CNN-PCA-SVM Hybrid Model	92.19% Accuracy
**PVEL-AD Dataset [20]**	10 (Various Internal Defects)	36,543	Deep Learning Object Detection	The first comparison provided
**SPHERE Dataset [23]**	3 (Broken, Clean, Dirty)	6079	YoloV8-m, YoloV9-e, Private CNN	97.26% Accuracy (YoloV8-m)
**Rahman Dataset [24]**	5 (Flawless, Dust, Flawed, Physical Damage, Scratches)	8973	YoLov11	mAP @0.5 of 85%
**UCF EL Failure Dataset [21]**	5 (Cracks, Contact Interruptions, Cell-Interconnection Failures, Contact Corrosion)	17,064	ResNet-50 via Deeplabv3	0.95 weighted F1-Score

The characteristics of the datasets in Table 1 are insufficient in terms of class imbalance or image count.

**Table 2:** Systematic analysis of the literature on photovoltaic cell defect datasets and methods.

Evaluation Criteria	Common Findings in the Literature	Observed Differences	Reasons for Differences
**Dataset Size and Diversity**	Most studies emphasize the importance of large datasets with various defect types to improve model generalizability and robustness [19,20,37,38]. Many datasets contain thousands to tens of thousands of images with multiple defect classes [20,21,37].	Some studies work with smaller datasets or specific subsets due to limited availability or focus; for example, datasets with only a few hundred images [19,39,40].	Differences arise from the availability of annotated data, intended use cases (e.g., production line vs. research), and imaging/acquisition methods that affect dataset size.
**Imaging Modalities**	EL imaging is primarily used due to its ability to clearly reveal micro-defects [19,28,41]. IR imaging is common, especially for thermal-related defects [42,43] and some studies combine modalities for richer data [44].	Some studies focus solely on one modality (e.g., only IR [37] or only EL [19]) or integrate limited modalities reflecting specific detection targets or equipment availability [44].	Variation stems from the targeted defect types, the cost and accessibility of imaging technologies, and the operational environment (laboratory/field).
**Class Distribution Balance**	There is a general consensus that class imbalance is a significant challenge in PV defect datasets. Many studies have addressed this by applying data augmentation (GANs, synthetic data) [42]. Some models apply class weighting strategies in their loss functions to reduce imbalance effects [45,46].	The scope and methods for addressing imbalance vary; some datasets inherently have more balanced class distributions, while others report serious imbalances requiring advanced augmentation [20].	Differences are related to the dataset source (general/proprietary), the prevalence of errors in real-world data, and the availability of sources for augmentation techniques.
**Annotation Detail Level and Standards**	Several studies facilitate semantic segmentation and object detection tasks by providing high-quality annotations, including pixel-level segmentation masks and bounding boxes [21,39,47]. Semi-supervised and few-shot learning approaches have also been investigated to reduce annotation effort [48].	Other datasets use coarser annotations, such as bounding boxes without image-level labels or segmentation masks [37,49]; annotation consistency varies depending on the challenges of manual labeling [50].	Discrepancies arise from annotation costs, the purpose of the dataset (classification vs. segmentation), and the availability of expert annotation. Semi-supervised methods help bridge these gaps.
**Data Augmentation Applications**	There is consensus on the use of data augmentation methods, including geometric transformations, GAN (Generative Adversarial Network)-based synthetic image generation, and contrast enhancement, to improve model robustness and address dataset limitations [42,51]. Scaling is particularly emphasized in small or unbalanced datasets [22].	There is variation in the types and scope of augmentation; some studies use simple augmentations (translation, rotation), while others use more advanced GANs or synthetic data generation [42].	Differences arise from variations in computational resources, dataset size, and the complexity of the defect features that models aim to capture.

**Table 3:** Summary of the eight defect classes.

Defect Class	Primary Cause	EL Image Characteristics	Expected Performance Impact
**Electrically Insulated Cell Parts**	Conductivity loss in soldered regions, ribbon discontinuities, poor soldering, corrosion, or mechanical stress	Dark segments along the connection line, localized brightness loss, asymmetric emission pattern	Weakening of current flow and reduced cell output; indicates poor electrical continuity
**Material Defect**	Structural inconsistencies related to wafer quality, diffusion process, or metallization deviations	Uneven brightness, cloudy regions, reduced contrast	May have limited visual effect in some cases, but can reduce current production and may be confused with other classes
**Finger Defect**	Breakage, thinning, or interruption of conductive fingers	Linear dark bands, discontinuous illumination along finger lines	Increased local resistance, weakened current collection, possible power loss and local heating
**Cell-Interconnection**	Severe structural damage, fragmentation, or breakage affecting interconnection paths	Disrupted cell regions, broken or separated structural appearance	Significant reduction in electricity generation, local power loss, increased thermal stress, and higher hot-spot risk
**Microcrack**	Thermo-mechanical stress during production, transport, assembly, or field operation	Thin branching lines, isolated dark islands, reduced emission in cracked regions	Initially limited impact, but performance degrades over time as crack growth reduces current-carrying area; may lead to hot spots
**Multi-Defect**	Simultaneous occurrence of two or more defects (e.g., Microcrack + Finger, Material + Connection Problem)	Overlapping linear defects, regional darkening, weakened connection lines	Greater performance loss than single-defect cases due to compound fault effects
**Visual**	Imaging or acquisition errors rather than physical cell damage (focus shift, blur, exposure error, sensor noise, shadowing, cropping)	Low contrast, blur, noisy appearance, shadowed or improperly framed cell image	No direct physical defect implication; included to prevent confusion between acquisition artifacts and real defects
**Normal**	No structural, electrical, or material defect	Uniform healthy EL appearance without abnormal dark regions	Represents normal electricity generation and healthy cell condition

**Table 4:** Class distributions obtained from the images.

Classes	Total Image Count
**Cell-Interconnection**	2538
**Electrically_Insulated_Cell_parts**	5000
**Finger**	5000
**Material**	5000
**Microcrack**	5000
**Multi-Defect**	5000
**Normal**	5000
**Visual**	5000

**Table 5:** Class-wise distribution of training and test images before and after data augmentation.

Classes	Test Images	Training Images Before Augmentation	Training Images After Augmentation	Final Total Images
**Cell-Interconnection**	508	677	2030	2538
**Electrically_Insulated_Cell_parts**	1000	1480	4000	5000
**Finger**	1000	4000	4000	5000
**Material**	1000	4000	4000	5000
**Microcrack**	1000	4000	4000	5000
**Multi-Defect**	1000	4000	4000	5000
**Normal**	1000	4000	4000	5000
**Visual**	1000	1450	4000	5000

**Table 6:** Table prepared by categorizing the 16 deep learning models used.

Category	Models
Classic CNN	AlexNet, VGG16, VGG19
Residual Networks	ResNet18/50/101
Dense Networks	DenseNet201
Inception-Based Models	GoogLeNet, InceptionV2, InceptionV3
Lightweight/Mobile	MobileNetV2, ShuffleNet, SqueezeNet
Scalable	EfficientNetB0
YOLO-Based Backbone	DarkNet19, DarkNet53

**Table 7:** Comparative information about the used deep learning models.

Architecture	Layers	Connections	Convolution Layers	Parameters	Memory (Mb)
**AlexNet [53]**	25	-	8	61 m	233
**Vgg16 [54]**	41	-	16	138 m	528
**Vgg19 [54]**	47	-	19	144 m	548
**GoogleNet [55]**	144	170	22	7 m	26.7
**Resnet18 [56]**	72	79	18	11.7 m	44.6
**ResNet50 [56]**	177	192	50	25.6 m	97.8
**Resnet101 [56]**	347	379	101	44.6 m	171
**SqueezeNet [57]**	68	75	18	1.24 m	4.7
**InceptionResnetv2 [58]**	825	922	164	55.9 m	213
**InceptionV3 [59]**	316	350	48	23.9 m	91.1
**MobileNetV2 [60]**	153	162	23	3.5 m	13.6
**DarkNet19 [61]**	64	63	19	20.8 m	79.6
**DarkNet53 [62]**	184	206	53	41.6 m	159
**DenseNet201 [63]**	709	806	201	20 m	77.3
**EfficientNetb0 [64]**	289	362	82	5.31 m	20.4
**ShuffleNet [65]**	171	186	50	1.4 m	5.5
**PVELNet**	132	151	26	1.79 m	7.98

**Table 8:** Hyperparameters used in training deep learning models.

Hyperparameter	Value	Information
Transfer learning	By changing the last layer	The original classification layer has been redefined according to the number of classes in the study.
Data splitting ratio	80% training—20% test	Randomized class-based partitioning.
Input size	Model-specific (224 × 224 × 3)	The images have been resized to the input size expected by the relevant model.
Optimization algorithm	SGDM	Stochastic Gradient Descent with Momentum.
Initial learning rate	0.0002	The initial step size of the training.
Learning rate schedule	Piecewise	The learning rate is reduced at specific epoch intervals.
Learning rate drop factor	0.001	LR = LR × 0.001 (each drop).
Learning rate drop period	Six epochs	The learning rate is reduced every six epochs.
Maximum epoch	10	Maximum number of passes over the training dataset.
Mini-batch size	64	Number of samples used in each iteration.
FC weight learning rate	10	Accelerated learning for the newly added fully connected layer.
FC bias learning rate	10	Accelerated learning for bias terms.
Training data shuffle	Randomized	Data shuffling is randomized at the beginning of each epoch.

**Table 9:** Evaluation metrics and their features used in the study.

Metric	Equation	Feature
**Precision**	$\frac{TP_{c}}{TP_{c}+FP_{c}}$	TP_c_: number of true-positives
**Recall**	$\frac{TP_{c}}{TP_{c}+FN_{c}}$	FP_c_: number of false-positives
**Accuracy**	$\frac{\sum_{c}TP_{c}}{N}$	FN_c_: number of false-negatives
**F1-Score**	$2\times \frac{Precision\times Recall}{Precision+Recall}$	It is calculated by taking the harmonic mean of the Precision and Recall values.

**Table 10:** Comparative data of the F1-Score values of 16 deep learning models and the proposed PVELNet model by class.

	F1-Score								
	Cell-Interconnection	Electrically Insulated Cell Parts	Finger	Material	Microcrack	Multi-Defect	Normal	Visual	Mean
DarkNet19	0.97	0.97	0.91	0.89	0.88	0.97	1.00	0.96	0.94
DarkNet53	0.98	0.97	0.91	0.91	0.90	0.97	1.00	0.96	0.95
MobileNetV2	0.95	0.96	0.87	0.89	0.86	0.97	1.00	0.92	0.93
ResNet18	0.97	0.97	0.88	0.89	0.87	0.96	0.99	0.93	0.93
ResNet50	0.94	0.96	0.87	0.91	0.83	0.97	1.00	0.97	0.93
ResNet101	0.96	0.97	0.90	0.88	0.89	0.97	1.00	0.96	0.94
Vgg16	0.97	0.98	0.90	0.91	0.90	0.98	1.00	0.94	0.95
Vgg19	0.96	0.98	0.91	0.91	0.91	0.97	0.99	0.94	0.95
SqueezeNet	0.95	0.96	0.88	0.88	0.87	0.97	0.99	0.94	0.93
GoogleNet	0.94	0.97	0.91	0.89	0.88	0.97	1.00	0.94	0.93
DenseNet201	0.96	0.97	0.89	0.90	0.88	0.97	1.00	0.94	0.94
AlexNet	0.96	0.96	0.90	0.90	0.88	0.96	1.00	0.93	0.94
InceptionV3	0.97	0.97	0.89	0.88	0.88	0.96	0.99	0.94	0.94
EfficientNetb0	0.91	0.95	0.84	0.85	0.80	0.96	0.98	0.91	0.90
InceptionV2	0.94	0.96	0.84	0.86	0.85	0.97	0.99	0.91	0.92
ShuffleNet	0.94	0.94	0.86	0.86	0.83	0.95	0.98	0.93	0.91
**PVELNet**	0.97	0.98	0.94	0.93	0.92	0.97	1.00	0.96	0.96

**Table 11:** Comparison of Recall values for 16 deep learning models and the proposed PVELNet model by class.

Recall									
	Cell-Interconnection	Electrically Insulated Cell Parts	Finger	Material	Microcrack	Multi-Defect	Normal	Visual	Mean
DarkNet19	0.97	0.97	0.89	0.95	0.85	0.97	1.00	0.96	0.94
DarkNet53	0.97	0.96	0.93	0.92	0.88	0.97	1.00	0.95	0.95
MobileNetV2	0.93	0.94	0.87	0.91	0.86	0.97	1.00	0.95	0.93
ResNet18	0.97	0.96	0.86	0.88	0.87	0.99	0.99	0.95	0.93
ResNet50	0.94	0.96	0.87	0.91	0.83	0.97	1.00	0.97	0.93
ResNet101	0.96	0.97	0.90	0.88	0.89	0.97	1.00	0.96	0.94
Vgg16	0.98	0.99	0.86	0.94	0.86	0.98	1.00	0.98	0.95
Vgg19	0.94	0.98	0.97	0.90	0.91	0.96	1.00	0.91	0.95
SqueezeNet	0.97	0.96	0.91	0.85	0.88	0.97	0.99	0.91	0.93
GoogleNet	0.90	0.98	0.92	0.88	0.87	0.97	1.00	0.95	0.93
DenseNet201	0.96	0.96	0.90	0.89	0.89	0.98	1.00	0.94	0.94
AlexNet	0.97	0.95	0.91	0.90	0.88	0.97	1.00	0.93	0.94
InceptionV3	0.97	0.96	0.89	0.88	0.88	0.97	1.00	0.94	0.94
EfficientNetb0	0.89	0.94	0.84	0.88	0.80	0.97	0.98	0.90	0.90
InceptionV2	0.96	0.96	0.81	0.86	0.85	0.97	1.00	0.94	0.92
ShuffleNet	0.95	0.94	0.85	0.84	0.86	0.99	0.97	0.92	0.91
**PVELNet**	0.97	0.97	0.94	0.93	0.92	0.98	1.00	0.96	0.96

**Table 12:** Comparison of Precision values for 16 deep learning models and the proposed PVELNet model by class data.

Precision									
	Cell-Interconnection	Electrically Insulated Cell Parts	Finger	Material	Microcrack	Multi-Defect	Normal	Visual	Mean
DarkNet19	0.97	0.98	0.93	0.84	0.92	0.97	1.00	0.96	0.95
DarkNet53	0.98	0.98	0.90	0.89	0.91	0.97	1.00	0.96	0.95
MobileNetV2	0.97	0.98	0.88	0.88	0.87	0.97	1.00	0.90	0.93
ResNet18	0.96	0.97	0.91	0.90	0.87	0.94	0.99	0.91	0.93
ResNet50	0.95	0.98	0.89	0.86	0.91	0.96	1.00	0.89	0.93
ResNet101	0.97	0.97	0.91	0.89	0.89	0.97	0.99	0.94	0.94
Vgg16	0.96	0.97	0.94	0.89	0.94	0.98	1.00	0.90	0.95
Vgg19	0.98	0.97	0.86	0.93	0.91	0.98	0.99	0.98	0.95
SqueezeNet	0.93	0.96	0.85	0.90	0.86	0.97	1.00	0.96	0.93
GoogleNet	0.98	0.95	0.90	0.89	0.89	0.96	0.99	0.93	0.94
DenseNet201	0.96	0.98	0.89	0.91	0.88	0.96	0.99	0.95	0.94
AlexNet	0.95	0.97	0.89	0.90	0.88	0.96	0.99	0.93	0.94
InceptionV3	0.97	0.97	0.90	0.88	0.88	0.96	0.99	0.94	0.94
EfficientNetb0	0.93	0.95	0.85	0.83	0.80	0.95	0.99	0.92	0.90
InceptionV2	0.91	0.97	0.87	0.87	0.85	0.97	0.99	0.88	0.91
ShuffleNet	0.93	0.95	0.87	0.89	0.81	0.92	0.99	0.94	0.91
**PVELNet**	0.98	0.98	0.93	0.93	0.92	0.97	1.00	0.95	0.96

**Table 13:** Classification accuracy and memory requirements of models.

	Model	Accuracy	Memory Space (MB)
**1**	DarkNet19	94.26	219
**2**	DarkNet53	94.65	446
**3**	MobileNetV2	92.61	36.8
**4**	ResNet18	93.14	125
**5**	ResNet50	92.83	267
**6**	ResNet101	94.02	478
**7**	Vgg16	94.58	1443
**8**	Vgg19	94.69	1505
**9**	SqueezeNet	92.77	13.3
**10**	GoogleNet	93.47	74.7
**11**	DenseNet201	93.90	232
**12**	AlexNet	93.49	650
**13**	InceptionV3	93.29	253
**14**	EfficientNetb0	90.02	59.2
**15**	InceptionV2	91.52	617
**16**	ShuffleNet	91.10	19.9
**17**	PVELNet	95.71	46.1

## Data Availability

The data presented in this study are available on request from the corresponding author. The data are not publicly available due to permissions and restrictions from the industrial data provider.
